# An update on the treatment of premature ejaculation: A systematic review

**DOI:** 10.1080/2090598X.2021.1943273

**Published:** 2021-08-04

**Authors:** Ramadan Saleh, Ahmad Majzoub, Mohammed Abu El-Hamd

**Affiliations:** a Department of Dermatology, Venereology and Andrology, Faculty of Medicine, Sohag University, Sohag, Egypt; b Urology Department, Hamad Medical corporation, Doha, Qatar; c Urology Department, Weill Cornell Medicine - Qatar, Doha, Qatar

**Keywords:** Male sexual dysfunctions, premature ejaculation, selective serotonin reuptake inhibitors, treatment of premature ejaculation, tricyclic antidepressants

## Abstract

To analyse the current therapeutic options for patients with premature ejaculation (PE) and highlight their mechanism(s) of action, effectiveness, advantages and limitations. A literature search was conducted using the PubMed database searching for articles exploring different PE treatment modalities. A Preferred Reporting Items for Systemic Reviews and Meta-Analyses (PRISMA) approach was used to report the results of the literature search. A total of 149 articles were included in this review. The currently available treatment methods for PE include behavioural therapy, local anaesthetics, tricyclic antidepressants, selective serotonin reuptake inhibitors, and selective phosphodiesterase inhibitors. Most PE treatments are either experimental or used off-label. New treatments are certainly warranted to overcome this exasperating sexual dysfunction.

**Abbreviations:** AIPE: Arabic Index of Premature Ejaculation; CNS: central nervous system; CYP: cytochrome P450; ED: erectile dysfunction; FDA: United States Food and Drug Administration; H1: histamine receptors; 5-HT: 5-hydroxytryptamine; IELT: The intravaginal ejaculation latency time; IPE: Index of Premature Ejaculation; M1: muscarinic receptors; OCD: obsessive–compulsive disorder; PDE5: phosphodiesterase type 5; PE: premature ejaculation; PEP: Premature Ejaculation Profile; PRO: patient-reported outcome; RCT: randomised controlled trial; SS: Severance Secret (cream); SSRIs: selective serotonin reuptake inhibitors; TCAs: tricyclic antidepressants

## Introduction

Premature ejaculation (PE) is perhaps the most common sexual dysfunction amongst men. The prevalence rate of PE is variable, but it is believed that one out of three men may complain of this sexual dysfunction at some point during their lives [1]. This disease entity has suffered from significant ambiguities in the past with respect to its definition and pathophysiology, and it was not until 2014 when the first standardised evidence-based definition of PE was established [2].

The evaluation of patients presenting with PE is initiated with a complete medical history looking for comorbidities that would make them prone to this clinical condition or would rather alter the offered treatment options (e.g. endocrine, urological, or psychorelational/psychosexual) [3,4] (Table 1). A detailed sexual history is obviously relevant to assess the frequency and nature of sexual encounters and to identify sexual comorbidities (e.g. erectile dysfunction [ED]) that would render PE simple (occurring in the absence of other sexual dysfunctions) or complicated (occurring in the presence of other sexual dysfunctions) [3]. The International Society for Sexual Medicine (ISSM) guidelines on PE recommends asking patients with such a presentation about the time between penetration and ejaculation (‘cumming’), their ability to delay ejaculation and the impact of such condition on their psychological wellbeing [5].

**Table 1.** The key steps for evaluation of patients with PE

Obtaining the patient’s general medical and sexual history. Classifying PE based on onset (e.g. lifelong or acquired), timing (e.g. prior to or during intercourse), and type (e.g. absolute/generalised or relative/situational). Involving the partner to determine their view of the situation and the impact of PE on the couple as a whole. Identifying sexual comorbidities (e.g. ED) to define whether PE is simple (occurring in the absence of other sexual dysfunctions) or complicated (occurring in the presence of other sexual dysfunctions). Performing physical examination to check the man’s sexual organs and reflexes. Identifying underlying aetiologies and risk factors (e.g. endocrine, urological, or psychorelational/psychosexual) to determine the primary cause of PE and any associated comorbidities. Discussing treatment options to find the most suitable intervention, according to the needs of the man and his partner.

It is also imperative to classify PE based on its onset into either lifelong or acquired PE and to assess the severity of the symptoms. Involving the partner during the initial and subsequent interviews is preferred to determine their view of the situation and the impact of PE and its treatment outcome on the couple as a whole. A genital examination is also recommended to evaluate the phallus and scrotal contents.

In addition, assessment of patients with PE includes the use of validated questionnaires and patient-reported outcome (PRO) measures (the ability to have control over ejaculation and the extent of patient and partner sexual satisfaction) in addition to stopwatch measures of ejaculatory latency. Stopwatch measures of intravaginal ejaculatory latency time (IELT) were widely used in clinical trials and observational studies of PE, but have not been recommended for use in routine clinical management of PE [6]. Despite the potential advantage of objective measurement, stopwatch measures have the disadvantage of being intrusive and potentially disruptive of sexual pleasure or spontaneity.

Five validated questionnaires have been developed and published to date. Two measures (Index of Premature Ejaculation [IPE] and Premature Ejaculation Profile [PEP]) have extensive databases. One measure (PE Diagnostic Tool) has a modest database. Two other measures (Arabic and Chinese PE Questionnaires) have few clinical trial data available [6].

Currently, no therapy is approved by the United States Food and Drug Administration (FDA) for treatment of PE [7–9]. However, several therapies for PE are marketed and used in many countries. Treatment modalities as recommended by the British Association of Sexual Health and HIV include behavioural therapy, tricyclic antidepressants (TCAs), selective serotonin reuptake inhibitors (SSRIs), local anaesthetic agents, and phosphodiesterase type 5 (PDE5) inhibitors [10] (Table 2). Numerous studies have shown that SSRIs and drugs with SSRI-like side-effects are safe and effective in the treatment of PE [11]. The aim of the present review was to explore the various therapeutic options available for PE and highlight their mechanism(s) of action, effectiveness, advantages, and limitations.

**Table 2.** The currently available treatment methods for PE

Behavioural therapy	Pharmacological therapy	Surgical therapy
1. Squeeze technique 2. Start/stop technique	1- Non-selective serotonin reuptake inhibitor antidepressants as: Tricyclic antidepressants (Clomipramine). 2- Selective serotonin reuptake inhibitors SSRIs antidepressants such as: a- Fluoxetine b- Citalopram c- Escitalopram d- Sertraline e- Paroxetine f- Fluvoxamine g- Dapoxetine 3- Topical therapy. a. Lidocaine-prilocaine 5% cream b. Local SS cream c. Lidocaine-prilocaine spray d. Dyclonine/alprostadil cream 4- PDE5 inhibitors 5- Opioid agonist. a. Tramadol 6- Others a. Intracorporeal Alprostadil b. Alpha adrenergic blockers c. Folic acid d. Caffeine e. Botulinum toxin injections	1. Glans augmentation 2. Dorsal neurectomy 3. Pulsed radiofrequency neuromodulation 4. Frenectomy 5. Surgical removal of foreskin remnants 6. Varicocelectomy

## Methods

### Search strategy

**Figure 1.**
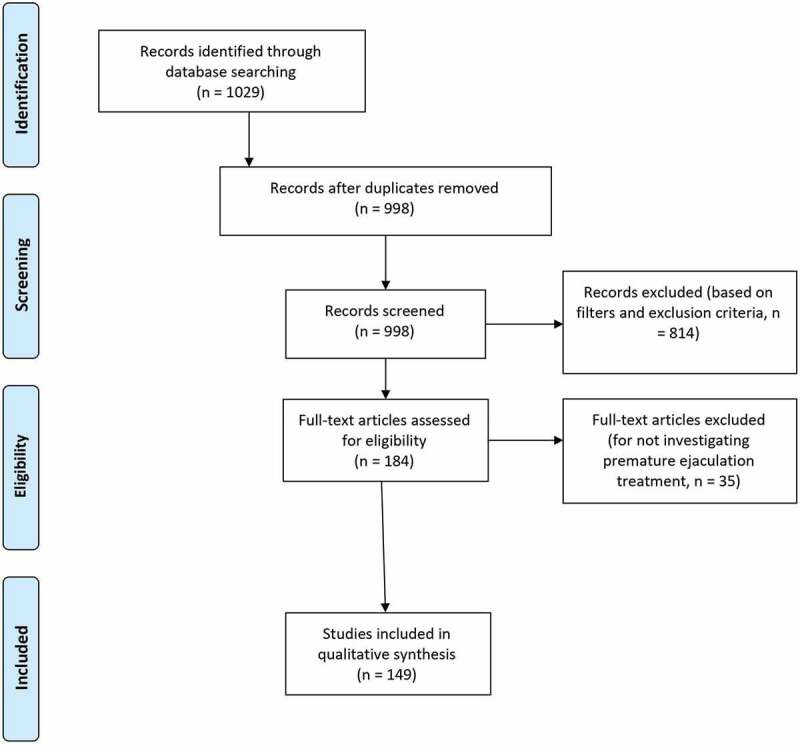
PRISMA 2009 flow diagram

This review was conducted according to Preferred Reporting Items for Systemic Reviews and Meta-Analyses (PRISMA) criteria. The PubMed database was searched using the key words ((‘premature ejaculation’)) AND ((‘treatment’ OR ‘management’)) from the time of its initiation until 10 January 2021. The following filters were applied to the searched results: (1) Humans, (2) English, (3) Male, and (4) Adults (aged >18 years). Screening of the searched articles’ titles, abstracts and main text was performed successively. Reviews, commentaries, editorials, abstracts, and case reports were excluded from this review. Articles not specifically designed to investigate a PE treatment modality were also excluded; these constituted studies exploring PE aetiology, epidemiology, pathophysiology, psychological impact etc. Relevant articles were selected for inclusion in the discussion of various PE treatment modalities in this review (Figure 1).

## Results

A total of 1029 articles were initially retrieved with the literature search, and 814 articles were excluded after removing duplicates and applying the search filters and exclusion criteria. Additionally, 35 articles did not meet the main objective of the search and were excluded leaving 149 studies that were included in the discussion of this manuscript.

## Discussion

Various treatment methods for PE have been described and include the following (Table 2).

### Behavioural therapy

The two most frequently used techniques in behavioural therapy are the ‘stop-and-start’ technique described by Semans in 1956 [12], and the ‘squeeze’ method developed by Masters and Johnson in 1970 [13]. These techniques were proven to be effective in most cases. However, couples can be averse to using them, with some women reluctant to squeeze their partner’s penis and some couples unwilling to interrupt sexual interaction once initiated [14]. These techniques focus on distraction and reduction of sexual excitement or stimulation, which may reduce overall sexual satisfaction.

#### 1. Squeeze technique

The man relaxes on his back and the wife starts to stimulate his penis. When the man indicates high arousal and orgasm is about to occur, the woman stops stimulation and applies a firm squeeze to the head of the penis for 5–10 s with the thumb on the frenulum and the index and middle fingers just above the coronal ridge on opposite side until the man feels that the ejaculation reflex is inhibited. The female repeats the technique again two or three times and then the man proceeds to ejaculation. After several times of this practice, the male will be more able to control his ejaculation and gains confidence.

The couple is instructed to start with the ‘woman-on-top’ position of intercourse. The man lies on his back and the wife sits on top of him. Once he has a firm erection, he inserts his penis into her vagina, with his hands on her thighs to guide her movement. He asks her to stop moving once ejaculation is about to occur. She can start moving when the man’s arousal subsides a little and so on.

The aim is to tolerate penetration without ejaculation for ~15 min. If the man is initially unable to do so, he should not worry; he just needs to repeat the exercise as often as he needs. Once he can control his ejaculation, the couple can make love in any position they like [13].

#### 2. Start/stop technique

This technique is more preferable and similar to the above one except that the female stops stimulation only and does not apply squeeze to the glans penis when the orgasm is about to occur. When the male assumes control with the hand of his partner, intercourse can be done, with the female on top, then lateral, and finally male on top position. At all times coitus is stopped near orgasm until control is possible [12].

### The goals of traditional psychotherapy/behavioural interventions

Psychotherapy/behavioural interventions improve ejaculatory control by helping men/couples to: (1) learn techniques to control and/or delay ejaculation, (2) gain confidence in their sexual performance, (3) lessen performance anxiety, (4) modify rigid sexual repertoires, (5) surmount barriers to intimacy, (6) resolve interpersonal issues that precipitate and maintain the dysfunction, (7) come to terms with feelings/thoughts that interfere with sexual function, and (8) increase communication [15–17].

### The effectiveness of the start/stop and squeeze techniques

Masters and Johnson [13] reported success rate of up to 98% of men with PE treated with the start/stop and squeeze techniques at a 5-years follow-up. This has often, erroneously, been translated into a success rate. It has been reported that only 64% of patients successfully gained ejaculatory control using the squeeze technique, and only one-third showed continued control for 3 years after treatment [18]. It was also found that men treated for PE using the same techniques experienced significant immediate benefits [19]. However, these gains were not sustained when measured at a follow-up visit 3 years later.

As it is not entirely clear why the intervention works in the first place, it is difficult to identify why the treatment gains were lost over time. Decrease in motivation, additional sexual problems occurring in the relationship, and changes in attraction between partners, could all play a role in the loss of gained ejaculatory control. Although, squeeze and start/stop techniques, have been the mainstay of PE management for many years, evidence of their short-term efficacy and long-term benefit is lacking [20].

*Pharmacological interventions (* Table 3 *) [24,26,27,32,47,48,50–52,55–59,66,67,72,73,76–79,81,84–87,89,90,116,151–163]*

**Table 3.** Studies investigating various antidepressants for the treatment of PE

Study	Methods	Intervention	Duration	Sample size	Outcome
Choi et al., 2019 [149]	RCT	Clomipramine 15 mg Placebo	12 weeks	159	Significant increase in IELT in treatment vs placebo, at a mean (SD) of 4.40 (5.29) vs 2.68 (2.03) min (*P* < 0.05) Significant improvement in PEDT score between both groups (p < 0.001).
Kim et al., 2018 [28]	RCT	Clomipramine 15 mg Clomipramine 30 mg Placebo	4 weeks	101	IELT of both the clomipramine 15 mg group and clomipramine 30 mg group was significantly increased 4 weeks after administration than the placebo group.
Waldinger et al., 2004 [27]	RCT	Clomipramine 25 mg Paroxetine 20 mg	4 weeks	30	Clomipramine led to a 4.05 (95% CI 3.26–5.02) fold-increase of the IELT. Paroxetine led to a 1.41 (95% CI 1.22–1.63) fold-increase of the IELT.
Segraves et al., 1993 [24]	RCT	Clomipramine 25 mg ↑ to 50 mg as needed Placebo	10 coital attempts	20	Average estimated time to ejaculation after vaginal penetration increased to 6.1 min on 25 mg of clomipramine and to 8.4 min on 50 mg of clomipramine.
Strassberg et al 1999., [26]	RCT	Clomipramine 25 mg Placebo	2 weeks	34; 23 with PE and 11 controls received treatment or placebo in 2-week phases	Increase in orgasmic latency in both groups; for PE: from <1 min when taking placebo to 3.5 min when taking clomipramine
Abdel-Hamid et al., 2001 [150]	RCT	Clomipramine 25 mg Sertraline 50 mg Paroxetine 20 mg Sildenafil 50 mg and pause-squeeze technique	4 weeks	31 randomised to receive the 5 treatments followed by 2-week wash-out between treatments.	Median IELT was significantly increased from the pre-treatment median of 1 min to 4, 3, 4, 15 and 3 min during treatment with clomipramine, sertraline, paroxetine, sildenafil and pause-squeeze technique, respectively (all *P* < 0.001). Sildenafil was superior to other modalities in terms of IELT and satisfaction (*P* < 0.001). The three antidepressants were comparable to each other in terms of efficacy (*P* > 0.05).
Jenkins et al., 2019 [47]	POS	Fluoxetine 20 mg	12 months	130	Self-rated ‘poor’ ejaculatory control decreased from 98–41% (*P* < 0.01), high personal distress from 47–11% (*P* < 0.01), and high partner distress rates from 72–27% (*P* < 0.01).
Kara et al., 1996 [46]	RCT	Fluoxetine 20 mg ↑ to 40 mg Placebo	4 weeks	17	The IELT noticeably increased after 4 weeks of treatment with fluoxetine, and patient recordings revealed that improvement began within 1 week of treatment. The mean (SD) intravaginal latency time before treatment was 25 (12.6) s in Group 1 and 30 (8.6) s in Group 2. The mean (SD) IELT increased to 180 (99.5) s in the treatment group (*P* < 0.05) and 60 (46.9) s in the placebo group (*P* > 0.05) 4 weeks after beginning treatment.
Manasia et al., 2003 [49]	RCT	Fluoxetine 90 mg/week Fluoxetine 20 mg/day	3 months	80	Mean (range) pre-treatment IELT for groups 1 and 2 was 0.48 (0–2.10) min and 0.50 (0–2.04) min, respectively. After 3 months of treatment of weekly and daily administration of fluoxetine the mean IELT was 3.57 and 3.37 min, respectively (*P* < 0.01). Partner sexual satisfaction and IIEF rate were greater with 90 mg fluoxetine, but no statistical difference was found.
Siroosbakht et al., 2019 [151]	RCT	Fluoxetine 20 mg Paroxetine 20 mg Citalopram 20 mg Sertraline 50 mg	8 weeks	480	Mean (SD) IELT before, 4 and 8 weeks after treatment in four groups was: sertraline 69.4 (54.3), 353.5 (190.4), 376.3 (143.5) s; fluoxetine 75.5 (64.3), 255.4 (168.2), 314.8 (190.4) s; paroxetine 71.5 (69.1), 320.7 (198.3), 379.9 (154.3) s; citalopram 90.39 (79.3), 279.9 (192.1), 282.5 (171.1) s, respectively. IELT significantly increased in all groups (*P* < 0.05), but there was no significant difference between the groups (*P* = 0.75).
Hosseini and Yarmohammadi, 2007 [50]	RCT	Fluoxetine 20 mg Sildenafil 50 mg	4 months	91, Group A: fluoxetine daily for 4 weeks then on demand for 4 months (*n* = 48) or Group B: same regimen + sildenafil for 4 months (*n* = 43)	Significant IELT improvement in both groups; Group A, from 0.5 to 4.3 min (*P* < 0.05); Group B, from 0.55 to 5.1 min (*P* < 0.005). No differences between both groups.
Mattos et al., 2008 [51]	RCT	Fluoxetine 90 mg Tadalafil 20 mg	4 weeks	90; 4 groups: 1) tadalafil plus fluoxetine, (2) fluoxetine plus placebo, (3) tadalafil plus placebo, and (4) two different placebo capsules (control).	Statistically significant difference in post-treatment IELT was seen with combined treatment compared to placebo (*P* < 0.001). There were increases in IELT from baseline in patients using fluoxetine plus tadalafil, from a mean (SD) of 49.57 (25.87) to 336.13 (224.77) s (*P* < 0.001); fluoxetine, from a mean (SD) of 56.55 (18.55) to 233.62 (105.08) s (*P* < 0.001); and tadalafil, from a mean (SD) of 49.26 (19.43) to 186.53 (159.05) s (*P* = 0.001).
Madeo et al., 2008 [57]	RCT	Fluoxetine 20 mg Citalopram 20 mg for 1 week then 40 mg Placebo	4 weeks	48	Increase in geometric IELT was observed in the citalopram (3 to 5.9 min), fluoxetine (3.1 to 4.1 min) and placebo groups (3.6 to 3.9 min). The increase was statistically significant in the citalopram group (*P* = 0.016)
Dadfar and Baghinia, 2010 [58]		Citalopram 20 mg	6 months	16 with failed fluoxetine treatment	The IELT and sexual satisfaction levels both significantly improved after citalopram prescription. The mean (SD) measured IVELT was 0.388 (0.212) min before the treatment, which increased to 4.313 (2.886) min after the treatment.
Kim and Seo, 1998 [152]	RCT	Fluoxetine 40 mg Sertraline 100 mg Clomipramine 50 mg Placebo	4 weeks	36; All patients took each drug and the placebo during a 4-week period per each agent with a washout period of at least 1 week between agents.	After 4 weeks of treatment with placebo, fluoxetine, sertraline and clomipramine the mean IELT was significantly increased from 46 s to 2.27 min, 2.30 min, 4.27 min and 5.75 min, respectively (all *P* < 0.01). However, treatment with clomipramine or sertraline caused a greater increase in mean IELT than fluoxetine or placebo (*P* < 0.01).
Akgul et al 2008 [56]	RCT	Sertraline 50 mg Citalopram 20 mg	8 weeks	80	Significant improvement was seen in both groups in terms of the IPE questionnaire results, from a mean (SD) pre-treatment score of 21.4 (1.8) to 39.8 (1.4) (*P* < 0.001) for citalopram group and from 20.9 (1.3) to 39.5 (2.9) (*P* < 0.001) for the sertraline group. However, the treatment response was not different between the two groups (*P* = 0.50).
Safarinejad and Hosseini, 2006 [55]	RCT	Citalopram 20 mg Placebo	12 weeks	58	The IELT after citalopram and placebo gradually increased from 32 and 28 s to ~268 and 38 s, respectively. The mean weekly intercourse episodes increased from pre-treatment values of 1.3 and 1.2 to 2.4 and 1.4, for citalopram and placebo, respectively (*P* < 0.05). Baseline mean intercourse satisfaction domain values of IIEF 10 and 11 reached to 16 and 10 at 12-week treatment in citalopram and placebo groups, respectively (*P* < 0.05).
Atmaca et al., 2002 [54]	RCT	Citalopram 20 mg ↑ to 60 mg Placebo	8 weeks	26	The increase in IELT in the citalopram group [week 0, mean (SD) 33.46 (17.9); week 8, 283.8 (80.5)] was statistically significant compared with the placebo group [week 0, 30.38 (14.6); week 8, 35.77 (13.5)] (*P* < 0.001).
Safarinejad, 2007 [65]	RCT	Escitalopram 10 mg Placebo	12 weeks	276	The escitalopram group had a 4.9-fold (95% CI 3.14–6.12) increase of the geometric mean IELT, whereas after placebo, the geometric mean IELT did not increase significantly (1.4-fold increase; 95% CI 0.86–1.68; *P* = 0.001).
Saleh et al., 2008 [66]	RCT	Escitalopram 10 mg Placebo	4 weeks	30	The mean (SD) score of IELT was significantly higher in escitalopram group as compared to placebo group after 30 days treatment, at 5.6 (0.7) vs 6.8 (0.4) (*P* < 0.03). At 90 days (2 months after stopping treatment), ILET score in the escitalopram group was significantly higher than the placebo group, at 6.7 (0.8) vs 3.4 (0.5) (*P* < 0.01).
Arafa and Shamloul, 2006 [71]	RCT	Sertraline 50 mg Placebo	4 weeks	147; crossover done after 1 week wash-out	Overall, 127 (81%) of 157 subjects had a significant increase in their AIPE total score after sertraline treatment. 66% of 100 patients available for follow-up had a relapse of PE within 6 months after sertraline withdrawal. IELT significantly improved in patients receiving sertraline vs placebo (*P* < 0.05)
Mathers et al., 2009 [153]	RCT	Sertraline 50 mg Vardenafil 20 mg	6 weeks	72; crossover done after 1 week wash-out	Initial mean (SD) PE scaled at 5.94 (1.6) and IELT was 0.59 min. Vardenafil improved PE grading: mean (SD) 2.7 (2.1) (*P* < 0.01) and IELT increased to 5.01 (3.69) min (*P* < 0.001). The mean (SD) PE grading improved 1.92 (1.32) (*P* < 0.01) and IELT 3.12 (1.89) min (*P* < 0.001) with sertraline.
Mendels et al., 1995 [154]	RCT	Sertraline 50 mg ↑ to 200 mg Placebo	8 weeks	52	Sertraline treatment produced significant improvements relative to placebo in time to ejaculation, at a mean (SD) of 1.16 (1.13) to 4.49 (2.9) vs 1.19 (1.38) to 2.46 (4.6) (*P* < 0.001); and number of successful attempts at intercourse, at a mean (SD) 0.58 (1.02) to 2.32 (2.4) vs 0.3 (0.47) to 0.75 (0.97) (*P* = 0.015), as well as overall clinical judgements of improvement.
Basar et al., 1999 [155]	RCT	Fluoxetine 20 mg for 1 week followed by 40 mg Sertraline 50 mg	4 weeks	57	In the fluoxetine group, 8 (30.8%) patients cured, improvement in 11 (42.3%) and failure in 7 (26.9%). In the sertraline group, 12 (38.7%) patients cured, improvement in 10 (32.3%) and failure in 9 (29%).
Xu et al., 2014 [72]	RCT	Sertraline 50 mg daily Mycelium of cordyceps sinensis C4	8 weeks	218; 63 patients chose to take sertraline 100 mg daily for an additional 4-week period, and 80 patients continued treatment with sertraline 50 mg.	Mean IELT of patients who subsequently chose to take 100 mg of sertraline was significantly lower than that of patients who continued taking 50 mg of sertraline, although the IELT value was comparable between the two groups of patients at baseline. However, with an additional 4-week treatment, the mean IELT increased significantly more in the 100-mg group than in the 50-mg continuation group.
McMahon, 1998 [31]	RCT	Sertraline 50 mg Placebo	4 weeks	37	The mean (range) pre-treatment IELT was 0.3 (0–1) min. The mean ejaculatory interval after 4 weeks of treatment was 3.2 min (range 1 min to anejaculation) with sertraline and 0.5 (0–1) min with placebo (*P* < 0.001).
Balbay et al., 1998 [156]	POS	Sertraline 50 mg	2 weeks	16	14 (87.5%) responded clinically. Clinical response was achieved in the first week of treatment in 11 of 16 responders (68.75%).
Abu El-Hamd and Abdelhameed, 2018 [88]	RCT	Paroxetine 30 mg Dapoxetine 30 mg Sildenafil 50 mg Placebo	6 weeks	150, 5 groups: placebo, paroxetine, dapoxetine, sildenafil and combined dapoxetine + sildenafil	The mean of IELT, satisfaction score and PEDT in all groups was significantly improved after treatment (*P* = 0.001). Combined dapoxetine with sildenafil group had the best values of IELT, satisfaction scores and PEDT in comparison with other treatment groups (*P* < 0.001).
Simsek et al., 2014 [89]	RCT	Dapoxetine (30 and 60 mg) Paroxetine (20 mg)	4 weeks	150, Group 1 were treated with on-demand dapoxetine (30 mg), Group 2 with on-demand dapoxetine (60 mg), and Group 3 with daily paroxetine (20 mg).	The IELT increased from baseline to post-treatment by 117%, 117% and 170% in the paroxetine group (*P* < 0.01), 30 mg dapoxetine group (*P* < 0.01) and 60 mg dapoxetine group (*P* < 0.01), respectively. The increase from baseline IELT were similar for the 30-mg dapoxetine and paroxetine groups (*P* > 0.05), while the 60-mg dapoxetine group had a larger post-treatment IELT increase compared with the 30-mg dapoxetine (*P* < 0.05) and paroxetine (*P* < 0.01) groups.
Waldinger et al., 1997 [75]	RCT	Paroxetine 20 mg Paroxetine 40 mg	7 weeks	27	Both groups showed a statistically significant difference from the baseline values of ejaculation latency (*P* < 0.001) and a clinically relevant improvement in ejaculation time. The increase in the IELT was not statistically significant different between the groups.
Alghobary et al., 2010 [78]	RCT	Paroxetine 20 mg Tramadol HCL 50 mg	12 weeks	35	After 12 weeks, a decline of IELT to fivefold was recorded with tramadol whereas further increase of IELT to 22-fold was recorded with paroxetine compared with baseline (*P* < 0.05). Tramadol improved AIPE score significantly after 6 weeks but not after 12 weeks vs baseline, whereas paroxetine increased the AIPE score after 6 and 12 weeks vs baseline (*P* < 0.05).
McMahon and Touma, 1999 [76]	PCS	Paroxetine 20 mg	4 weeks	94; Group A, once daily followed by on demand (*n* = 64) Group B, on demand (*n* = 33)	The mean (range) pre-treatment IELT of both Group A and B was 0.4 (0–1) min. In Group A, the mean ELT was 4.5 min (range 1–anejac.). 53/61 men in Group A regarded their ejaculatory control as improved and were then treated with ‘on-demand’ paroxetine, achieving an ELT of 3.9 min (range 0–10). 63 men in this group of 53 regarded that they had maintained improved ejaculatory control with a mean ELT of 5.5 min (range 2–20 min) after a further four weeks of treatment (*P* < 0.001). The remaining 17 men reported a recurrence of poor ejaculatory control with a mean ELT of 0.7 min (range 0–2 min). In group B with initial ‘on-demand’ paroxetine after a mean of 4.5 weeks of treatment, the mean ELT was 1.5 min (range 0–5 min).
Safarinejad, 2006 [77]	RCT	Dapoxetine 60 mg (Group 1, *n* = 115) Paroxetine 20 mg (Group 2, *n* = 113) Placebo (Group 3, *n* = 112)	12 weeks	340	At the end of the 12-week treatment with dapoxetine, paroxetine, and placebo, the mean IELT was increased from 38, 31 and 34 s to 179, 370 and 55 s, respectively (*P* = 0.01 in Group 1 and *P* = 0.001 in Group 2).
Salonia et al., 2002 [115]	RCT	Paroxetine 10–20 mg Sildenafil 50 mg	6 months	80; Group 1 – Paroxetine 10 mg daily then 20 mg on demand. Group 2 – Paroxetine 10 mg daily then 20 mg on demand + sildenafil 50 mg on demand	Mean (SE) IELT in Group 1 was 0.33 (0.04), 3.7 (0.10) (*P* < 0.01) and 4.2 (0.03) min (*P* < 0.01) at baseline, 3 and 6-month follow-up, while in Group 2 it was 0.35 (0.03), 4.5 (0.07) (*P* < 0.01) and 5.3 (0.02) min (*P* < 0.001), respectively. When improvement in IELT was compared in the two groups, Group 2 results proved to be significantly greater (*P* < 0.05).
Polat et al., 2014 [157]	RCT	Paroxetine 20 mg Tadalafil 20 mg	4 weeks	150; Group 1 – paroxetine daily for 1 month, Group 2 – tadalafil on demand, and Group 3 – paroxetine and tadalafil on demand	Statistically significant changes in IELT were detected in comparison to baseline results [mean (SD) Group 1: 60.6(30.2) to 117.3 (67.3) s, Group2: 68.5 (21.4) to 110.2 (37.3) s, Group 3: 71.56 (40.23) to 175.2 (60.2) s (*P* < 0.01). IELT scores after discontinuation of treatment were found to be close to the baseline IELT scores (*P* > 0.05).
Waldinger et al., 1998 [80]	RCT	Fluoxetine 20 mg Fluvoxamine 100 mg Paroxetine 20 mg Sertraline 50 mg Placebo	6 weeks	51	During the 6-week treatment period, the geometric mean IELT in the placebo group was constant at ~20 s. Analysis of variance revealed a between-groups difference in the evolution of IELT delay (*P* < 0.001); in the paroxetine, fluoxetine, and sertraline groups there was a gradual increase to ~110 s, whereas in the fluvoxamine group, IELT was increased to only ~40 s. The paroxetine, fluoxetine, and sertraline groups differed significantly (*P* < 0.001, *P* < 0.001, *P* = 0.017, respectively) from placebo but the fluvoxamine group did not (*P* = 0.38).
Pryor et al., 2006 [86]	RCT	Dapoxetine 30 mg Dapoxetine 60 mg Placebo	12 weeks	1958	Dapoxetine significantly prolonged IELT (*P* < 0.001, all doses vs placebo). Mean (SD) IELT at baseline was 0.90 (0.47), 0.92 (0.50), and 0.91 (0.48) min, and at study endpoint (week 12 or final visit) was 1.75 (2.21) min for placebo, 2.78 (3.48) min for 30 mg dapoxetine, and 3.32 (3.68) min for 60 mg dapoxetine.
Kaufman et al., 2009 [84]	RCT	Dapoxetine 60 mg Placebo	9 weeks	1238	Personal distress related to ejaculation decreased from a mean (SD) of 2.8 (0.81) to 1.5 (1.05) in treatment group vs 2.8 (0.82) to 2 (1.05) in the placebo group. Perceived control over ejaculation improved from a mean (SD) of 0.6 (0.61) to 2.1 (1.13) in the treatment group vs 0.6 (0.59) to 1.6 (1.02) in the placebo group. Satisfaction with sexual intercourse improved from a mean (SD) of 1.4 (0.83) to 2.5 (1.11) in the treatment group vs 1.5 (0.79) to 2(1.01) in the placebo group.
Buvat et al., 2009 [83]	RCT	Dapoxetine 30 mg Dapoxetine 60 mg Placebo	24 weeks	618	Mean average IELT increased from 0.9 min at baseline (all groups) to 1.9, 3.2, and 3.5 min with placebo and dapoxetine 30 mg and dapoxetine 60 mg, respectively, at study end point; geometric mean IELT increased from 0.7 min at baseline to 1.1, 1.8, and 2.3 min, respectively, at study end point. All PEP measures and IELTs improved significantly with dapoxetine vs placebo at week 12 and week 24 (all *P* < 0.001).
McMahon et al., 2010 [85]	RCT	Dapoxetine 30 mg Dapoxetine 60 mg Placebo	12 weeks	858	Mean Average IELT increased from ~1.1 min at baseline (across groups) to 2.4, 3.9, and 4.2 min with placebo, dapoxetine 30 mg, and dapoxetine 60 mg, respectively; and geometric mean IELT increased from ~0.9 min at baseline (across groups) to 1.8, 2.7, and 3.1 min, respectively (fold-increases of 2.0, 2.8, and 3.3, respectively). All PEP measures and the CGI of change were significantly improved with dapoxetine vs placebo at study endpoint (*P* < or = 0.005 for all).
McMahon et al., 2013 [158]	RCT	Dapoxetine 30 mg Dapoxetine 60 mg Placebo	12 weeks	429	Arithmetic mean average IELT significantly increased with dapoxetine vs placebo at end point (5.2 vs 3.4 min) and weeks 4, 8, and 12 (*P* ≤ 0.002 for all). Men who described their PE at least ‘better’ using the CGI were significantly greater with dapoxetine vs placebo at end point (56.5% vs 35.4%) and weeks 4, 8, and 12 (all *P* ≤ 0.001). Significantly better outcomes were also reported with dapoxetine vs placebo on PEP measures.
Tuken et al., 2019 [159]	POS	Dapoxetine/Sildenafil combination 30/50 mg	4 weeks	53	The geometric mean (SD) IELT of the patients significantly increased from 22.72 (15.16) to 68.25 (82.33) s (*P* < 0.001). Similarly, significant improvements were observed in the mean (SD) PEP index score [0.86 (0.72) vs 2.36 (1.13); *P* < 0.001) and IIEF-EF domain score [13.17 (3.33) vs 24.60 (3.96); *P* < 0.001). According to the GIC results, 81.13% of the patients were satisfied with the treatment.
Peng et al., 2020 [160]	POS	Dapoxetine 30 mg	4 weeks	154	An obvious improvement compared with the baseline was found regarding mean (SD) IELT [2.4 (1.6) vs1.0 (0.7) min; *P* < 0.001) and mean NITBE [85.9 (61.9) vs 37.4 (28.6) times; *P* < 0.001). The proportion of patients with a self-evaluation of at least ‘slightly better’ and were categorised into ‘CGIC ≥1ʹ group was 70.1%.

CGI: Clinical Global Impression-Improvement scale; NITBE: number of intravaginal thrusts before ejaculation; POS: prospective observational study.

#### 1. Tricyclic antidepressants (TCAs)

All TCAs have a three-ring nucleus in their molecular structures [21]. TCAs, and their derivatives, have been a cornerstone in medical treatment of depression. They are very effective, but, their use is often associated with a variety of unpleasant and sometimes dangerous side-effects [22] (Table 4) [29,74,75,80,83,164–174]. Unwanted effects of TCAs arise through interactions with several different neurotransmitter systems.

**Table 4.** List of the dosage, pharmacokinetics and side-effects of commonly prescribed TCAs and SSRIs for PE

Drug	Dosage, mg	Metabolism	Half life	Side-effects
Clomipramine [,^79^]	25	CYP2C19, CYP3A4, CYP1A2	10–70 h	Manic episode in both bipolar and unipolar disorders. Anticholinergic side-effects: dry mouth, blurring of vision, constipation, urinary retention and aggravation of narrow-angle glaucoma. Cardiac side-effects including tachycardia, flattened T waves, prolonged QT intervals, and depressed ST segments
Fluoxetine [161,162]	20–40 daily	CYP1A2, CYP2C9, CYP2C19, CYP2D6, CYP3A4	1–4 days	Dizziness, dry mouth, nausea, constipation, weight gain, insomnia, tremors, prolonged QT interval, light headedness, confusion, agitation, sexual dysfunction
Citalopram [163,164]	20	CYP2C19, CYP2D6, CYP3A4	35 h	
Escitalopram [165–168]	10	CYP2C19, CYP3A4	35 h	
Sertraline [79]	25–50	CYP1A2, CYP2C9, CYP2C19, CYP2D6, CYP3A4	26 h	
Paroxetine [73,74,169]	20	CYP1A2, CYP2C9, CYP2C19, CYP2D6, CYP3A4	Variable depends on dose	
Fluvoxamine [79,170]	50–300	CYP1A2, CYP2C9, CYP2C19, CYP2D6, CYP3A4	15 h and 17 to 22 h	
Dapoxetine [82,171]	30 or 60	CYP2D6, CYP3A4	1.4 and 20 h	

### Clomipramine

Clomipramine is a TCA that is indicated for the treatment of major depressive episodes, secondary depression, panic disorder with agoraphobia, generalised anxiety disorder, and obsessive–compulsive disorder (OCD) [23]. Low doses of clomipramine have been suggested to be effective for the treatment of PE [24].

### Mechanism of action and efficacy of clomipramine in the treatment of PE

The mechanism by which clomipramine delays ejaculation is unclear. Decreased reuptake of serotonin (or 5-hydroxytryptamine [5-HT]) has been suggested as a mechanism by which several antidepressants including clomipramine delay ejaculation. Clomipramine may increase the sensory threshold for the stimuli in the genital area [25].

Administration of 25 mg clomipramine orally 4 h before planned sexual intercourse was effective in the treatment of patients with PE [26,27]. However, the drug may cause mild yet annoying non-sexual side-effects such as sleepiness and yawning on the day of coitus, and significant nausea the day after. On-demand use of 15 mg clomipramine orally 4 h before sexual intercourse for 4 weeks was safe and effective in prolonging IELT in the treatment of patients with PE [28].

### Drug interactions

The antihypertensive effect of propranolol and clonidine may be blocked by clomipramine. Clomipramine with α-methyldopa may cause behavioural agitation. The plasma levels of clomipramine and antipsychotics are increased by their co-administration. The sedation effect of clomipramine and opioids, alcohol and hypnotics are increased by their co-administration [].

#### 2. Selective serotonin reuptake inhibitors (SSRIs)

The SSRIs have emerged as an effective treatment for patients with PE whether or not these patients suffer from depression [29]. They were especially indicated in cases of failed or rejection of psychological treatment, and when partners were unwilling to cooperate in treatment. The SSRIs are widely used because of their safety, tolerability and demonstrated efficacy across a broad range of clinical conditions [11].

The ability of SSRIs to delay ejaculation was first coincidentally discovered as a result of use of these drugs in the treatment of depression in men in the 1970s [30]. The basic principle is that serotonin is a central inhibitory neurotransmitter for sexual function whereas dopamine is a central excitatory neurotransmitter. Antidepressants either elevate serotonin levels leading to inhibition of genital reflexes, or decrease dopamine with the same results [31].

All SSRIs inhibit reuptake of serotonin into presynaptic serotonergic neurones, an action that increases the availability of serotonin at the synapse and ultimately, enhances serotonergic function in the central nervous system (CNS). This mechanism of action depends on the binding of the drug to serotonin transporter protein [32]. It has also been suggested that the efficacy of SSRIs in inhibiting PE, is probably due to increase synaptic 5-HT concentrations via blockade of the 5-HT transporter and activation of the 5-HT 2 C receptor, which then decreases the function of the 5-HT 1A receptor or restores the balance between the two receptor functions (5-HT 1A and 5-HT 2 C) [33,34].

### Methods of administration of SSRIs

#### 1. Acute SSRI administration

The 5-HT transporter blockade induced by acute administration of all current SSRIs leads to higher serotonin levels in the synapse and in the space around the cells [35]. Increasing serotonin levels activates 5-HT1A auto-receptors, resulting in less serotonin being released into the synaptic cleft within minutes [36]. A higher serotonin concentration increases activation of presynaptic 5-HT1B auto-receptors, which alone can reduce the release of serotonin.

**Figure 2.**
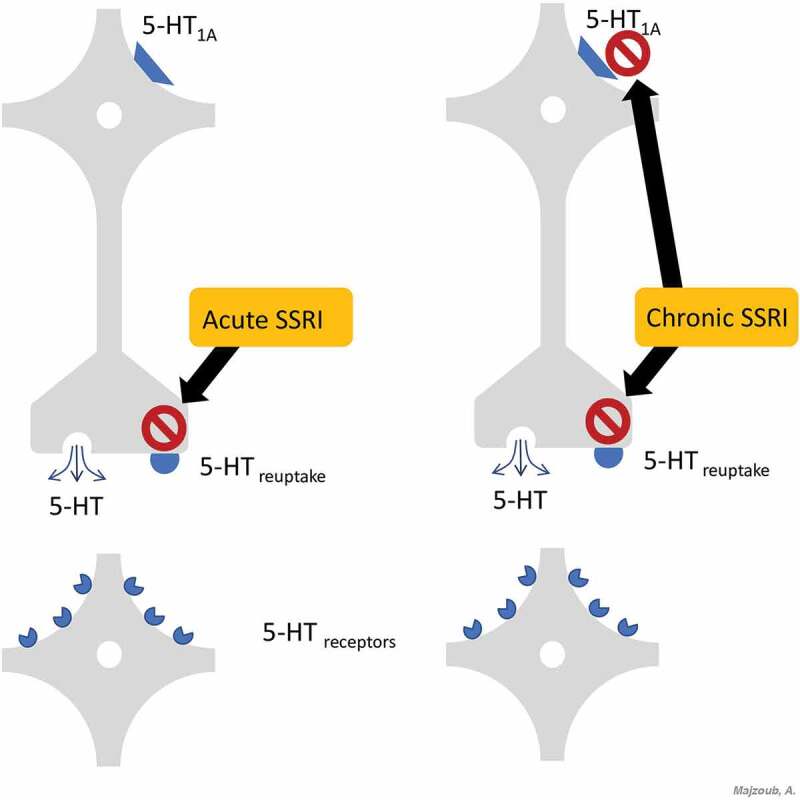
Mechanism of action of SSRIs at the synaptic terminal

Under normal physiological conditions, the net effect of acute administration of SSRIs is little to no increase in serotonin neurotransmission and minimal or no stimulation of postsynaptic 5-HT receptors (Figure 2). Given this background, on-demand SSRI treatment would not be expected to result in acute stimulation of 5-HT postsynaptic receptors. Consequently, one would expect minimal increase in synaptic serotonin levels and, thus, little or no synaptic stimulation of 5-HT receptors. Little or no activation of postsynaptic 5-HT receptors should then result in no clinically relevant delay of ejaculation [37].

#### 2. Chronic SSRI administration

In contrast to their acute administration, chronic use of currently available SSRIs causes some physiological changes that delay ejaculation. Ongoing blockade of 5-HT receptors that mediate serotonin reuptake results in a persistent increase in serotonin levels in the synapse and in the space around the cells (Figure 2). As opposed to the acute administration of SSRIs, this ongoing blockage leads to desensitisation of the 5-HT1A auto-receptors in a few weeks; and possibly of the 5-HT1B auto-receptors [38].

The net effect of the chronic administration of SSRIs is an increase in the serotonin released into the synapse and enhanced serotonin neurotransmission, thus resulting in a stronger activation of the 5-HT postsynaptic receptors compared with that observed in acute SSRI administration [38]. These data predict that daily treatment of SSRI will stimulate the 5-HT postsynaptic receptors, leading to a clinically relevant ejaculation delay after 1–2 weeks of continuous intake [37].

### Limitations associated with of SSRIs

While serotonergic drugs are extensively used for the treatment of PE, there are limitations associated with their use. Some of these limitations include unwanted sexual side-effects such as decreased sexual desire and ED [39]. A sudden reduction or cessation of long-term treatment with an SSRI can lead to ‘SSRI discontinuation syndrome’, a group of physical and psychological symptoms including nausea, vomiting, dizziness, headache, ataxia, drowsiness, excitement, anxiety, and insomnia. These symptoms begin 1–3 days after drug cessation and typically continue for >1 week. These side-effects were reversible with SSRI re-introduction [40].

An SSRI overdose or interaction with other drugs, can enhance serotonin activity in the CNS to the point of causing the ‘serotonin syndrome’, a group of serious, persistent symptoms including myoclonus, hyperreflexia, sweating, shivering, discoordination, and mental status changes [41].

### Fluoxetine

Fluoxetine is the parent drug of all SSRIs. It has largely (albeit not completely) substituted older and less safe drugs such as TCAs. Fluoxetine is a serotonin-specific antidepressant approved in 1987 by the FDA for treatment of depression [42]. It is also a treatment option for patients with Alzheimer’s disease who have severe obsessive–compulsive symptoms [43] and for patients with intention myoclonus [44].

### Efficacy of fluoxetine in treatment of PE

Fluoxetine is more selective and more potent in retarding ejaculation as compared to TCAs [45]. At a dose of 20 mg daily for 1 week followed by 40 mg daily for ~4 weeks, fluoxetine successfully improved PE in a double-blind placebo controlled study of 17 patients [46]. Another study revealed that a significant decrease in self-reported ‘poor’ ejaculatory control, high personal distress and high partner distress were noted in men receiving 20 mg fluoxetine for 12 months [47].

The rationale with which fluoxetine is thought to exhibit its beneficial effects is through increasing the penile sensory threshold, without changing the amplitudes and latencies of sacral evoked response and cortical somatosensory evoked potentials [48]. A study compared 90 mg once weekly dose with 20 mg daily doses fluoxetine on 80 patients with PE [49]. After a 4-month treatment period, the authors reported significant prolongation in the IELT, together with improved International Index of Erectile Function (IIEF) results and partner sexual satisfaction in both groups. There were no significant differences between both treatment methods in terms of efficacy and reported side-effects.

The co-administration of fluoxetine and PDE5 inhibitors appears to have a potentiating effect on sexual satisfaction. The combination of fluoxetine (20 mg fluoxetine daily for 4 weeks followed by 20 mg on-demand 2–3 h before planned sexual activity for 4 months) with sildenafil (50 mg 1 h before sexual activity for 4 months) resulted in significantly better IELT and intercourse satisfaction compared with fluoxetine alone in patients with PE [50]. Similarly, administration of 90 mg fluoxetine once per week plus 20 mg tadalafil within 36-h before planned sexual intercourse for 12 weeks in patients with lifelong PE resulted in significantly longer IELT compared with fluoxetine only or tadalafil only treatment [51].

### Citalopram

Citalopram is a potent specific inhibitor of neuronal serotonin reuptake [52] that is indicated for the treatment of depression, anxiety, panic disorder, OCD, premenstrual dysphoria, alcohol dependence, and behavioural disturbances of dementia [53].

### Efficacy of citalopram in treatment of PE

The daily administration of 20 mg citalopram in patients with PE resulted in significant increase in IELT, improved overall patient sexual satisfaction, and decreased performance anxiety [54,55]. Citalopram was compared to SSRIs in several studies. A randomised controlled trial (RCT) compared the efficacy of citalopram 20 mg to sertraline 50 mg for a treatment period of 8 weeks in patients with PE. The authors reported a statistically significant increase in the results of the IPE questionnaire in both the citalopram and sertraline groups, without a significant difference in efficacy between the two treatments. No serious adverse effects were detected in any of the patients and both drugs were well tolerated [56].

A randomised, placebo-controlled, double-blind study evaluated the effects of 20 mg/day citalopram and 20 mg/day fluoxetine [57]. The authors assessed the effect of the intervention on masturbation IELT, Rigiscan, and the IIEF-15 questionnaire. Results revealed a delay in the IELT in both the treatment groups compared with placebo; however, the difference was only statistically significant for the citalopram group. No significant effect on nocturnal penile tumescence, measured with Rigiscan, was observed in any of the treatment groups.

The authors of the latter study concluded that while there was no objective effect on penile erection, the subjective sexual impairment could be attributed to the significant delay in ejaculation achieved with citalopram). Another study evaluated IELT and sexual satisfaction in 16 newly married men with PE, and a history of unsuccessful treatment with fluoxetine, using citalopram as a salvage treatment [58]. The IELT was significantly improved after treatment with citalopram together with a significant increase in sexual satisfaction

### Escitalopram

Escitalopram is the S-isomer of the racemic compound citalopram, that is widely used in both psychiatric and primary care practices for the treatment of depression. It was found to be effective and well tolerated in treating depression at a dose of 10 mg/day [59,60]. At this dose, escitalopram is at least as effective as citalopram 40 mg/day [59]. Escitalopram also has been shown to be rapidly effective in treating symptoms of anxiety associated with depression [61].

Escitalopram is the most selective molecule for serotonin receptors compared to other antidepressants [62]. In a radio-ligand binding study of cells expressing human serotonin transporters, escitalopram proved to be ~30-times more potent than its enantiomer, R-citalopram, in its capacity to bind to the serotonin transporter receptor site [32].

Escitalopram was more selective for serotonergic transport proteins when compared with other SSRIs such as fluoxetine, paroxetine, fluvoxamine or sertraline [32]. Escitalopram had little or no binding affinity for >100 binding sites tested *in vitro*, including α-adrenergic (α1) receptors, muscarinic (M1) receptors and histamine (H1) receptors [63]. Selectivity for serotonergic, rather than muscarinic, histaminergic, or adrenergic receptors suggested a lower potential for causing dry mouth, sedation, or cardiovascular side-effects [32,62]. Patients who have switched from fluoxetine, sertraline, paroxetine, or citalopram to escitalopram due to adverse events experienced a lower incidence of side-effects [64].

### Efficacy of escitalopram in treatment of PE

A double-blind placebo-controlled study assessing the efficacy of daily administration of 10 mg escitalopram in 276 patients with PE for 12 weeks revealed a significant 4.9-fold increase in the IELT in the treatment group together with significant increase in intercourse satisfaction (measured with the IIEF-15) [65]. Similar results were re-demonstrated by another study of similar design showing that the effect of escitalopram was well maintained up to 2 months after stopping the drug [66]. A study compared the efficacy of three SSRIs (escitalopram 10 mg/day, fluoxetine 20 mg/day and paroxetine 20 mg/day) on subjective PE symptoms of 100 men measured using the Arabic Index of Premature Ejaculation (AIPE) [67]. The authors reported a significant improvement in PE symptoms after treatment, without a statistically significant difference in efficacy between the three treatments.

### Sertraline

Sertraline is another SSRI that is used for a number of conditions, including major depressive disorder, OCD, body dysmorphic disorder, and panic disorder [68]. Sertraline is a potent inhibitor of serotonin reuptake and is ~14-times more potent than fluoxetine in enhancing serotonergic neurotransmission [69].

### Efficacy of sertraline in treatment of PE

Several studies have shown that sertraline is an effective agent in the treatment of PE, indicated by increased IELT and partner’s sexual satisfaction [31,70]. A daily dose of 25–50 mg for 2–3 weeks is recommended after which the drug can be taken on-demand, 4–6 h before sexual activity [31]. A prospective placebo-controlled crossover study of 147 men with PE receiving sertraline 50 mg/day or placebo for 4-week intervals demonstrated significant improvement in the IELT and sexual satisfaction, using the AIPE, only in patients receiving sertraline treatment [71].

Another study compared the efficacy of sertraline 50 mg/day vs citalopram 20 mg/day for 8 weeks in 80 patients with PE [56]. The authors documented significant increase in IPE results in both treatment groups without any significant difference in the efficacy of the two agents. No serious adverse effects were detected in any of the patients and both drugs were well tolerated. A recent study suggested that increasing the dose of sertraline to 100 mg/day in patients not responding to an 8-week treatment with sertraline 50 mg/day demonstrated good tolerance to the higher dose regimen [72].

### Paroxetine hydrochloride

Paroxetine is an antidepressant SSRI that has been shown to be effective in treatment of major depression, panic disorder, OCD, and social anxiety disorder [73,74].

### Efficacy of paroxetine in treatment of PE

Paroxetine in a dose of 20 mg was an adequate treatment for primary PE, and a further increase in ejaculation latency may be achieved by increasing the dose [75]. The effect of paroxetine on the prolongation of the IELT after 6 weeks of treatment was significantly better if patients were treated initially with 20 mg paroxetine daily for 2 weeks, followed by 4 weeks of on-demand dosing compared with patients who began on-demand dosing with 20 mg [76]. Compared with other medications used for the treatment of PE, administration of 20 mg/day paroxetine for 12 weeks in patients with PE resulted in comparable efficacy to dapoxetine 60 mg/day with a statistically significant increase in the IELT and intercourse satisfaction domains of the IIEF-15 [77].

Paroxetine 20 mg/day was also compared to tramadol HCl 50 mg on-demand in 35 patients with lifelong PE who were randomised to receive either treatment and were followed-up after 6 and 12 weeks of therapy [78]. Paroxetine and tramadol increased IELT significantly by 11-fold and sevenfold after 6 weeks of therapy, respectively. However, after 12 weeks, further increase in the IELT to 22-fold was only detected in patients receiving paroxetine. Changes in the IELT were demonstrated by the AIPE results, as patients receiving paroxetine reported significant improvement after 6 and 12 weeks of therapy, while those receiving tramadol reported a significant improvement after 6 weeks and not 12 weeks of therapy

### Fluvoxamine

Fluvoxamine is a potent specific inhibitor of neuronal serotonin reuptake with few side-effects [79]. It appears to be effective in treatment of depression, OCD, and PE [22].

### Efficacy of fluvoxamine in treatment of PE

Fluvoxamine is probably the least effective SSRI in the treatment of PE. A double-blind, placebo-controlled study was conducted in men with lifelong PE to compare the efficacy of four SSRIs (fluoxetine, fluvoxamine, paroxetine, and sertraline) in delaying ejaculation [80]. Patients were randomised to receive fluoxetine 20 mg/day, fluvoxamine 100 mg/day, paroxetine 20 mg/day, sertraline 50 mg/day, or placebo for 6 weeks. A significant increase in the IELT was only observed in the paroxetine, fluoxetine, and sertraline groups, whereas in the fluvoxamine group, a minimal increase in the IELT was detected.

### Dapoxetine

Dapoxetine is the first member of the SSRI family to be developed specifically for the treatment of PE [81]. Its rapid absorption and elimination in the body makes it suitable for the treatment of PE, but not as an antidepressant [82].

### Efficacy in treatment of PE

A systemic review of five randomised, placebo-controlled phase III clinical trials [83–86] including 4232 men with PE from 32 countries confirmed that dapoxetine 30 and 60 mg increased the IELT and improved PROs of control, ejaculation-related distress, interpersonal distress and sexual satisfaction compared with placebo [87]. The pooled data revealed a statistically significant increase in the IELT with dapoxetine 30 mg (+2.3 min), dapoxetine 60 mg (+2.7 min) compared with placebo (+1.5 min). Dapoxetine 30 and 60 mg were well tolerated with a low incidence of severe adverse effects.

A recent single-blind placebo-controlled clinical study compared the efficacy of on-demand treatment with paroxetine 30 mg, dapoxetine 30 mg, sildenafil 50 mg, combined dapoxetine 30 mg + sildenafil citrate 50 mg and placebo on 150 patients with PE for a period of 6 weeks [88]. The authors reported significant improvement in the IELT, sexual satisfaction score and PE diagnostic tool score in all groups after treatment, with the best values reported in the combined dapoxetine + sildenafil citrate group. Another comparative study assessed the efficacy of on-demand dapoxetine 30 mg, on-demand dapoxetine 60 mg and daily paroxetine 20 mg on 150 patients with PE for a 1-month duration [89]. The IELT increased by 117%, 170% and 117% from baseline to post-treatment in the 30 mg dapoxetine group, 60 mg dapoxetine group and paroxetine group; respectively. Dapoxetine was found to have an additive effect on psychotherapy for the treatment of patients with PE.

### SSRI drug interactions

Almost all SSRIs are dependent on liver metabolism by cytochrome P450 (CYP450) enzyme isoforms each with a distinct profile of inhibition (Table 4). When SSRIs are co-administered with drugs that are metabolised by a specific CYP450 enzyme, they compete for binding to the active site of the enzyme [90], inhibiting the metabolism of the other drug substrates and elevating their plasma levels. This prolonged drug action can place patients at increased risk of drug toxicity. This drug interaction is often faced when SSRIs are co-administered with psychiatric drugs, as well as other medications given for a variety of medical conditions.

The SSRIs are not uncommonly combined with TCAs to treat psychiatric patients. As both drug classes are metabolised by CYP450 enzymes, the resulting interaction is well documented and increased plasma concentrations of TCAs have been reported. More specifically, concomitant use of fluoxetine or paroxetine, both potent CYP2D6 inhibitors, with TCAs was shown to be associated with up to fivefold increase in plasma concentrations of TCAs, and patients undergoing this interaction exhibited toxicity symptoms such as sedation, dry mouth, and urinary retention [91–93]. Fluvoxamine, a potent CYP2C19 inhibitor, caused up to fourfold increases in plasma concentrations of the TCAs amitriptyline, imipramine, and clomipramine and related clinical signs of toxicity [92].

Antipsychotics are another example of drugs that interact with SSRIs as they are metabolised by one or more of the CYP450 enzymes. Clozapine, olanzapine, and risperidone are the antipsychotics with the most frequent reported effects [94,95]. Fluoxetine and fluvoxamine can interact with anticonvulsants valproate and carbamazepine through their inhibitory effect on CYP2C9 and CYP3A4/5 respectively. Serious reactions including excessive tiredness, irritability, dizziness, and tremor have been reported leading to the discontinuation of the SSRI [96,97].

Serotonin syndrome is a group of symptoms that may occur following the combination of two or more serotonergic medications. It is typically seen when SSRIs are co-administered with lithium, tryptophan and monoamine oxidase inhibitors [98]. The release of serotonin by platelets is important for maintaining haemostasis, thus SSRIs may increase the risk of bleeding.

Interactions have been reported with anticoagulants such as warfarin and clopidogrel. Based on their strong inhibition of CYP2C9, fluoxetine and fluvoxamine have the highest potential risk for inhibiting warfarin metabolism thereby causing excessive bleeding. Furthermore, several SSRIs are potent inhibitors to CYP2C19 abolishing the antiplatelet response to clopidogrel [99].

### Topical therapy (anaesthetics)

#### A. Lidocaine-prilocaine 5% cream

Topical anaesthetics have been used for treatment of PE to decrease penile stimulation, and thus delay the time to ejaculation. The application of lidocaine-prilocaine 5% cream to penis before covering it with a condom 20–30 min before intercourse delayed ejaculation in men with PE. Prolonged application (30–45 min) before intercourse resulted in loss of erection [100]. Reduction in genital sensitivity of both partners may limit repeated use of topical anaesthetics [101].

Lidocaine-prilocaine cream used for a period of 30–60 days significantly increases the mean IELT, especially when penile hypersensitivity is likely to be the cause. The main side-effects of topical anaesthetic application included retarded ejaculation of >30 min, decreased penile sensitivity, penile irritation, and decreased vaginal sensitivity [102]. Topical anaesthetics are contraindicated for patients and/or their partners with allergies to any component of the product [103]. Administration of 20 mg fluoxetine daily plus local application of lidocaine ointment was found to be more effective than fluoxetine alone [104].

#### B. Local Severance Secret (SS) cream

The SS-cream is formed from nine natural substances including ginseng and cinnamon and has local desensitising and vasoactive effects differing from the local anaesthetics in the fact that it persists for up to 2 h. It promises to be an effective and safe therapeutic modality for patients with PE [105]. Applying SS-cream on the glans penis 1 h before planned sexual intercourse, lead to prolongation of ejaculatory latency time and improvement of sexual satisfaction for both partners with no adverse effect [105,106]. A study assessed penile vibratory threshold change using a bio-thesiometer using various doses of SS-cream and found that SS-cream increased the penile sensory threshold in a dose-dependent manner [107].

#### C. Lidocaine-prilocaine spray

Topical eutectic mixture for PE (TEMPE or PSD502) is a formulation of lidocaine and prilocaine in a metered dose aerosol-delivery system. Each spray delivers 7.5 mg lidocaine and 2.5 mg prilocaine. It is fast acting (within 5 min) and appears to be effective in improving the IELT and sexual satisfaction in patients with PE. It does not penetrate keratinised epithelium, and so only anaesthetises the glans, with no systemic side-effects and a low incidence of local side-effects [108,109].

#### D. Dyclonine/alprostadil cream

Dyclonine is a local anaesthetic usually used in the field of dentistry. It has been combined with the vasodilator alprostadil and used to treat PE. The product is applied 5–20 min before intercourse to the tip of the penis in the region of the meatus. One pilot study claimed positive results with it; however, the data were limited and further studies are warranted before conclusions can be made [110].

### Phosphodiesterase type 5 inhibitors

Several clinical trials have examined the potential effectiveness of the PDE5 inhibitor, sildenafil, in the treatment of PE. The rationale for the use of PDE5 inhibitors in the treatment of PE may be due to peripheral and/or central mechanisms. Ejaculation retarding by peripheral actions may include modulation of contractile response of vas deferens, seminal vesicles, prostate and urethra, induction of a state of peripheral analgesia, and prolongation of the total duration of erection. Central mechanisms may involve lessening of the central sympathetic output [111].

The on-demand administration of 50–100 mg sildenafil citrate for 1–3 months, in patients with PE complicated by ED, was found to safely and effectively improve erectile function and prolong ejaculation [112]. In addition, sildenafil citrate increased confidence, perception of ejaculatory control, and overall sexual satisfaction, and decreased the refractory time to achieve a second erection after ejaculation in men with PE [113]. It has also been shown that sildenafil combined with behavioural therapy produced more prolongation of the IELT and better male and female satisfaction than behavioural therapy alone in the treatment of patients with PE [114]. On-demand administration of 50 mg sildenafil 1 h before planned sexual activity combined with 10 mg paroxetine daily for 3 weeks and then 20 mg on demand, for 6 months provided significant increases in the ILET and intercourse satisfaction than paroxetine alone in potent patients with PE. However, combined treatment is associated with a mild increase in drug-related side-effects (headache and flushing episodes) [115].

Sildenafil citrate combined with paroxetine and psychological and behavioural counselling alleviated PE in patients in whom other treatments failed [116]. Administration of 50 mg sildenafil on-demand 1 h before planned sexual activity combined with 50 mg sertraline daily, for 12 weeks produced more prolongation of the IELT, and better male and female satisfaction than sertraline alone in patients with PE [117]. Furthermore, the on-demand administration of 50 mg sildenafil citrate 1 h before planned sexual activity for 6 months in patients with PE provided significant increases in the IELT and intercourse satisfaction than 20 mg paroxetine daily and the squeeze technique. However, adverse effects were more frequent in the sildenafil and paroxetine groups compared to the squeeze technique group. The most frequent adverse effects for sildenafil were headache and nasal congestion [118].

Other forms of PDE5 inhibitors have been used for the treatment of PE. The administration tadalafil 20 mg within 36 h before planned sexual intercourse combined with fluoxetine 90 mg once per week for 12 weeks in patients with lifelong PE resulted in significant increase in the IELT when compared to fluoxetine or tadalafil alone [51]. The on-demand administration of 10 mg vardenafil for 16 weeks provided significant increase of IELT and reduced post-ejaculatory refractory time in men with lifelong PE [119]. Improvement in confidence, perception of ejaculatory control and overall sexual satisfaction were reported.

The use of 5 mg tadalafil once daily plus lidocaine anaesthetic spray in treatment of lifelong PE was more effective than tadalafil alone or lidocaine anaesthetic spray alone [120]. In a single- blind placebo-controlled clinical study, the combined use of on-demand dapoxetine (30 mg) with sildenafil (50 mg) for patients with PE for 6 weeks had the best values of IELT and satisfaction scores in comparison with dapoxetine alone or sildenafil alone [88].

### Opioid agonist

Tramadol is a centrally acting analgesic with two mechanisms of action. It exerts an effect on the μ-opioid receptor, but also inhibits noradrenaline and serotonin reuptake. Its mechanism of action in PE is poorly understood; however, it is thought to be related to its action on the μ-opioid receptor, which may reduce sensitivity, as well as the inhibition of serotonin reuptake, which may delay ejaculation. It has a safety profile that is well tolerated with few side-effects [121].

Administration of 50 mg tramadol HCl 2 h before planned sexual activity for 8 weeks, resulted in a significant increase of IELT and intercourse satisfaction vs placebo [55]. Administration of 25 mg tramadol, 1–2 h before planned sexual activity for 8 weeks, significantly increased the IELT and intercourse satisfaction [121]. Administration of 50 mg tramadol with behavioural modification, 2 h before planned sexual activity for 8 weeks resulted in significant improvement in the IELT and overall sexual satisfaction [122]. On-demand administration of 62 mg tramadol (oral disintegrating tablet), 2–8 h before planned sexual intercourse for 12 weeks in patients with mild to severe PE resulted in significant increase in the IELT, improvement in overall sexual satisfaction and control over ejaculation, and decreased ejaculation-related personal distress and interpersonal difficulty [123].

As tramadol is an opioid, with weak µ-opioid activity, there are concerns about its abuse and dependence. Long-term studies are needed to determine the verifiable risk of opioid addiction [9,124].

### Surgical treatments

#### a. Glans augmentation

Glans augmentation has been a technique proposed to desensitise the glans penis and slow the ejaculatory reflex. It is a method in which hyaluronic acid is injected into the glans at the coronal edge to provide analgesia of the penis. Hyaluronic acid is a glycosaminoglycan and bulking agent that has been used to insulate the nerve endings and provide long-term (>1 year) local anaesthesia. It was reported to increase the IELT and satisfaction in patients with PE [125,126].

#### b. Dorsal neurectomy

Dorsal neurectomy with or without glandular augmentation with hyaluronic acid gel has been reported for treatment of refractory PE. It showed a significant increase in the IELT and patient satisfaction but associated with significant side-effects, including penile numbness, paraesthesia and pain [125]. It has been reported that selective neurotomy of the dorsal penile nerve preserved potency and decreased sensitivity [127].

#### c. Pulsed radiofrequency neuromodulation

Pulsed radiofrequency neuromodulation has been used for treatment of PE by desensitisation of the dorsal penile nerves. It showed a significant increase in the IELT in patients with PE. There were no reported side-effects post-procedure such as pain, penile hypoesthesia, or ED [128].

#### d. Frenectomy

Frenectomy has been studied as a possible surgical therapy for PE because of the association of PE with a short frenulum, or frenulum breve [129]. This condition can be congenital resulting from defects during sexual development or acquired secondary to excessive scarring occurring after rupture of a normal frenulum [130]. It hinders complete retraction of the prepuce during erection causing a ventral curvature of the glans. It is thought that a short frenulum may illicit PE through two plausible premises: (1) an uncomfortable sense of traction during intercourse that may trigger the end of coitus, (2) being a reservoir of nerve endings that are directly exposed to the tactile stimulation during intercourse [129].

#### e. Surgical removal of foreskin remnants

Surgical removal of foreskin remnants in incomplete circumcised adult patients with PE resulted in a significant increase in the IELT, overall sexual satisfaction, and control over ejaculation because it significantly decreased hypersensitivity of penis [131].

#### f. Varicocelectomy

A higher prevalence of PE has been reported in men with varicocele for unclear reasons [132,133]. Some have postulated that an increase in local genital temperature or the resulting androgen disruption that occurs with varicocele could be possible explanations [134]. Several studies have clearly reported an improvement in PE and testicular hormonal function in patients following varicocele ligation [135,136]. However, such an indication for varicocelectomy is not yet supported by any of the international guidelines of male reproduction.

### Other treatment

A. Adrenergic nerve blockade has been proposed as a treatment for PE. A clinical trial showed modest efficacy with alfuzosin and terazosin [137]. Silodosin, a highly selective α_1A_-adrenoceptor antagonist and on-demand use of 4 mg silodosin orally 1 h before sexual intercourse in treatment of patients with PE was effective in improving PE profile and the IELT [138]. The treatment was based on the fact that emission and ejaculation are under the influence of the sympathetic nervous system [139].

B. Folic acid provides the methyl group for the conversion of methionine to S-adenosylmethionine, which itself has been shown to influence serotonin metabolism. It has an important role in the synthesis of tetrahydrobiopterin, the rate limiting step in the synthesis of dopamine, noradrenaline and serotonin [140,141]. Folic acid supplementation was reported to produce an antidepressant-like effect, mediated by an interaction with the noradrenergic receptors (α_1_ and α_2_) and serotonergic receptors (5-HT1A and 5-HT2A/2 C) [142]. Low folate is associated with poorer response to SSRIs. Folate deficiency is associated with decreased serotonin activity [143] and folate supplementation increases cerebrospinal fluid levels of 5-hydroxyindoleacetic acid (the main metabolite of serotonin) in folate deficient patients suffering from depression [144]. Therefore, folic acid was suggested to exert a significant role in the pathogenesis of PE. Folic acid administration produces anti-PE-like effects dependent on the 5-HT systems. Folic acid may offer a cheaper, safer, more efficacious and more acceptable alternative to the conventional SSRIs for men with PE. However, more information is needed about the dosage, possible side-effects, and populations suited for the therapy [122].

C. Caffeine is a recently proposed treatment for PE. A double-blind RCT found that using 100 mg caffeine 2 h before intercourse signiﬁcantly improved the IELT and sexual satisfaction of 40 patients with PE [145]. Being a purine alkaloid, caffeine is a CNS stimulant and can increase the levels of multiple neurotransmitters including dopamine and serotonin [146].

D. A RCT using rat models, injections of botulinum toxin into each bulbospongiosus muscle increased the IELT relative to the group with saline injections. There was no effect on the rats’ ability to achieve and maintain an erection [147]. Mechanism would likely rely on the toxin’s ability to paralyse the neural end-plate, decreasing the ability of the muscles associated with ejaculation to contract [148].

## Conclusions

Premature ejaculation is a commonly encountered male sexual dysfunction, with a potential negative impact on the patient and his partner. In the present review, we explored the different therapeutic approaches that are currently available for the treatment of PE including behavioural, pharmacological, and surgical options. Interestingly, the medications currently used in the treatment of PE are sold off-label. Except for the newly licensed dapoxetine, which provides an effective, on-demand treatment regimen with relatively minimal side-effects, it is not clear whether it received final FDA approval for treatment of PE. Therefore, it is important for the clinician to recognise all PE treatment options as each patient may respond differently and experience variable side-effects. Future efforts should be directed towards understanding the exact pathophysiology of PE at different clinical setups and developing additional therapies with higher efficacy and minimal or no adverse effects.
